# A comparison of bolus injection of landiolol versus oral administration of propranolol before cardiac computed tomography

**DOI:** 10.1186/2193-1801-3-93

**Published:** 2014-02-17

**Authors:** Yoshitaka Nakamura, Kyohei Yamaji, Tatsunori Saho, Zyousin Matsuzaki, Itsuo Yuda, Yoshimitsu Soga, Shinichi Shirai, Kenji Ando, Masakiyo Nobuyoshi

**Affiliations:** Division of Cardiology, Kokura Memorial Hospital, 3-2-1 Asano, Kokurakita-ku, Kitakyushu, Japan; Division of Radiological Technology, Kokura Memorial Hospital, 3-2-1 Asano, Kokurakita-ku, Kitakyushu, Japan

**Keywords:** Cardiac computed tomography, Landiolol, Propranolol, Image quality, Coronary artery disease

## Abstract

Heart rate (HR) reduction is essential to achieve good image quality for cardiac computed tomography (CCT). We evaluated the efficacy of a bolus injection of landiolol, an ultra-short acting β-blocker, without the administration of oral β-blocker to reduce HR prior to CCT. We enrolled 678 consecutive patients who underwent CCT from December 2011 to March 2012 and divided them into three groups, which were a propranolol group (n = 277), a low-dose landiolol group (n = 188), and a high-dose landiolol group (n = 213). Patients in the propranolol group received oral propranolol (10–20 mg) prior to CCT. Patients in the low-dose and high-dose landiolol groups were administered a bolus injection of landiolol (0.125 mg/kg), while the high-dose group received an additional 3.75 mg of landiolol if the baseline HR was ≥75/min. Although the average HR was significantly lower in the propranolol group (61.6 ± 8.0/min) than in the low-dose landiolol group (64.1 ± 7.4/min, P < 0.001), there was no significant difference in the image quality (P = 0.91). Among patients with baseline HR ≥75/min, the average HR tended to be lower in the high-dose landiolol group (67.2 ± 6.9/min) compared with the low-dose landiolol group (69.0 ± 6.9/min, P = 0.10), and there was a corresponding difference in image quality between these two groups (P = 0.02). In conclusion, Although the decrease of HR was significantly larger in the propranolol group than in the landiolol groups, the image quality was similar. Among the patients who received landiolol, a higher dose was associated with a lower HR and better image quality. Further investigation to assess higher-dose bolus injection of landiolol or bolus injection following oral administration of a β-blocker would be needed.

## Background

With the recent development of multidetector computed tomography (MDCT), cardiac computed tomography (CCT) has become a reliable noninvasive diagnostic tool for coronary artery disease. (Achenbach et al. 2000; Nieman et al. 2001; Miller et al. 2008; Budoff et al. 2008; Meijboom et al. 2008; Raff et al. 2005). However, heart rate (HR) reduction is essential to achieve adequate image quality for diagnostic purposes, despite the recent advances in CT scanner hardware that have achieved a maximal rotation time of 270 to 350 msec. (Leschka et al. 2006; Shim et al. 2005; Muenzel et al. 2011; Korosoglou et al. 2010; Khan et al. 2011). Thus, either oral or intravenous β-blockers are routinely administered prior to CCT in order to obtain adequate images. (Gerber et al. 2003; Shapiro et al. 2008; Pannu et al. 2008; Degertekin et al. 2008; Roberts et al. 2009) Isobe et al. reported that continuous infusion of landiolol, a highly cardioselective and ultra-short acting β-blocker, was useful for CCT.(Isobe et al. 2008). Recently, Osawa et al. reported that bolus injection of landiolol following oral administration of metoprolol achieved sufficient reduction of the heart rate. (Osawa et al. 2013). Both of these initial studies included a relatively small number of patients. Therefore, we conducted a larger study to evaluate the efficacy of a single bolus injection of landiolol without prior oral premedication before CCT. The objective of the current study was to clarify whether the bolus injection of ultra-short acting β-blocker could improve the image quality or not.

## Methods

### Study population

**Figure 1:**
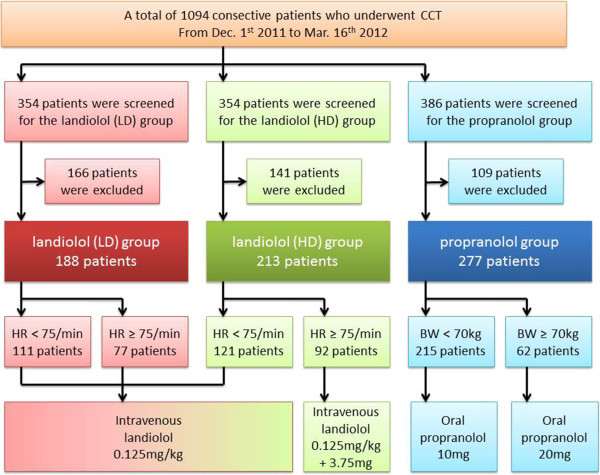
**Study flow chart.** There was a low-dose landiolol group, a high-dose landiolol group, and a propranolol group. CCT, cardiac computed tomography; LD, low dose; HD, high dose; HR, heart rate; BW, body weight.

A flow chart of the study is shown in Figure 1. A total of 1,094 consecutive patients who underwent CCT from December 1, 2011 to March 16, 2012 at our hospital were enrolled in this study. From December 1, 2011 to January 22, 2012 oral propranolol (Inderal, AstraZeneca K.K., Osaka, Japan) was administered before CCT (propranolol group), whereas intravenous landiolol hydrochloride (Corebeta, Ono Pharmaceutical Co., Osaka, Japan) was injected before CCT from January 23, 2012 to March 16, 2012. The patients who received landiolol were divided into two groups: one was given a low dose of landiolol from January 23 to February 18 (low-dose landiolol group) and the other was given a high dose of landiolol from February 19 to March 16 (high-dose landiolol group). Exclusion criteria were as follows: CCT for evaluation of bypass grafts or the ascending aorta, HR before administration <60/min or >90/min, atrial fibrillation, implanted pacemaker, a history of vasospastic angina, advanced atrioventricular block, left ventricular ejection fraction <40%, systolic blood pressure <90 mmHg, and known drug allergy. During the enrollment period for the propranolol group, patients who had a history of bronchial asthma were also excluded. All patients gave written informed consent and this study was approved by the hospital’s institutional ethics committee.

### Landiolol groups

Landiolol was injected as an intravenous bolus after initial scanning in the supine position and CCT was performed from 3 to 5 minutes later. A bolus dose of 0.125 mg/kg of landiolol was administered to patients in the low-dose landiolol group. In the high-dose landiolol group, the same dose of landiolol was administered to patients with a baseline HR <75/min (n = 121, 56.8%), while both 0.125 mg/kg and an additional 3.75 mg of landiolol were administered to patients with an HR ≥75/min (n = 92, 43.2%). The total dose of landiolol was limited to 12.5 mg in both groups. The low-dose and high-dose landiolol groups were analyzed on an intention-to-treat basis.

### Propranolol group

In the propranolol group, premedication with oral propranolol (10 mg) was given at 1.5 hours before CCT for patients with a body weight <70 kg, while 20 mg was administered for patients with a body weight ≥70 kg. Additional intravenous administration of β-blockers was prohibited by the study protocol.

### CT scanning and post-processing

Patients received premedication with nitroglycerin at 0.3 mg sublingually, unless this was contraindicated. Scanning was performed using a 64-row MDCT scanner (Light Speed VCT, GE Healthcare. Waukesha, Wisconsin) with 64 × 0.625-mm collimation and a gantry rotation time of 350 msec. The tube current was modulated with a maximum of 550 to 700 mA (depending on the patient’s size) during the period between 40% and 80% of the R-R interval and reduction by 80% during the remainder of the cardiac cycle, while the tube voltage was fixed at 120 kV. The helical pitch was selected just before scanning from 0.16 to 0.24 depending on the heart rate: helical pitch of 0.16 for patients with HR <36/min, 0.18 for HR <40/min, 0.20 for HR <44/min, 0.22 for HR <48/min, 0.24 for HR <62/min, and 0.16 for HR ≥62/min. A lower helical pitch was selected in patients who were likely to have premature beats during the main scan, whereas a higher pitch was selected in young patients and patients who had difficulty with breathholding. Final selection of the helical pitch was at the discretion of the radiologist. A test bolus of iohexol (Omnipaque 350 350 mgI/ml, Daiichi Sankyo, Tokyo, Japan) or iopamidol (Iopamiron 370 370 mgI/ml, Bayer Yakuhin, Osaka, Japan) was administered at a rate of 2.4 ml/sec to 5.0 ml/sec to ascertain the optimum rate and timing of injection for the main scan. Then 30 ml to 80 ml of contrast medium was injected intravenously during the main scan and a saline bolus (20 ml) was injected intravenously immediately after the contrast agent (double-bolus protocol). For post-processing, the optimum time window was selected for each coronary vessel from either mid-diastole (70, 75, or 80%) of the cardiac cycle, or end-systole (40, 45, or 50%). The “segment” and “burst” algorithms were used for half-scan reconstruction and multisector reconstruction, respectively. The optimal reconstruction algorithm was selected by viewing all images reconstructed with both algorithms side by side. The average and range of the HR during the main scan, total radiation exposure, main scan duration, occurrence of premature beats, and failure to maintain breathholding during the scan were recorded.

### Data analysis

**Figure 2:**
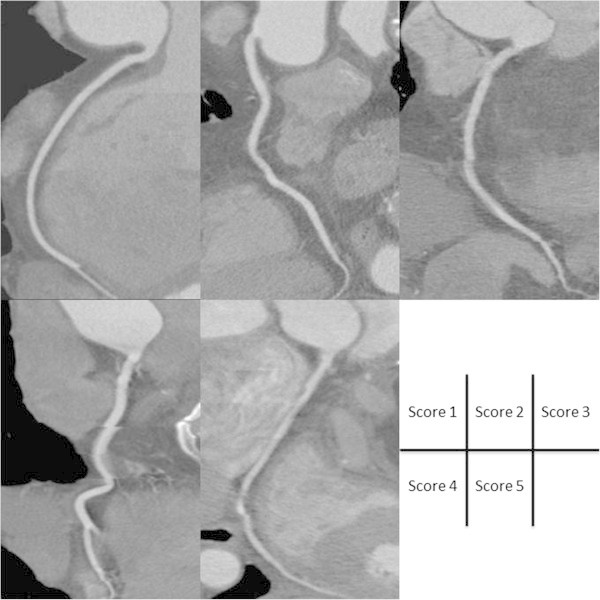
**Curved multiplanar reconstruction images obtained by CCT.** Examples of images given each score on five-point scale.

Assessment of the image quality score for each coronary vessel was done by two independent radiologists in a blinded manner regarding the clinical background using a previously reported five-point scale. (Leschka et al. 2006; Shim et al. 2005). (1) no motion artifacts and clear delineation of the segment; (2) minor artifacts and mild blurring of the segment; (3) moderate artifacts and moderate blurring without discontinuity; (4) severe artifacts and overlap or discontinuity of the segment; and (5) image not evaluable and vascular structures indistinguishable (Figure 2). The image quality score for a patient was defined as the maximum image quality score among the vessels.

### Statistical analysis

Categorical variables and numerical variables were expressed as numbers and percentages. Frequency analysis was performed with the *χ*^*2*^ test. Variables such as the image quality score were compared using the Kruskal-Wallis test. Normality of distribution was tested with the Shapiro-Wilk test. Continuous variables were expressed as the mean ± SD and were compared by the unpaired *t*-test. To compare patient characteristics among the 3 groups, one-way analysis of variance was used. Variation in the decrease of HR was assessed by the Bartlett’s test. All statistical analyses were performed using JMP 9.03 software (SAS Institute, Cary, NC). All reported P values are two-sided and P < 0.05 was regarded as statistically significant.

## Results

From a total of 1,094 consecutive patients who underwent CCT, 188 patients, 213 patients, and 277 patients were enrolled in the low-dose landiolol group, the high-dose landiolol group, and the propranolol group, respectively, while 416 patients met the exclusion criteria and were excluded as shown in Table 1. There was no significant difference in age, gender, height, weight, body mass index, and HR before β-blocker administration among the groups (Table 2).

**Table 1:** **Exclusion criteria**

	Low-dose landiolol	High-dose landiolol	Propranolol
	n = 166/354	n = 141/354	n = 109/386
CCT for evaluating bypass graft or ascending aorta	51 (14.4%)	40 (11.3%)	19 (4.9%)
HR <60/min before administration	69 (19.5%)	59 (16.7%)	37 (9.6%)
HR >90/min before administration	16 (4.5%)	24 (6.8%)	18 (4.7%)
Atrial fibrillation	14 (4.0%)	14 (4.0%)	9 (2.3%)
Permanent pacemaker	7 (2.0%)	3 (0.8%)	3 (0.8%)
History of vasospastic angina	2 (0.6%)	0 (0.0%)	1 (0.3%)
Advanced atrioventricular block	2 (0.6%)	1 (0.3%)	0 (0.0%)
History of asthma	0 (0.0%)	0 (0.0%)	20 (5.2%)
LVEF <40%	3 (0.8%)	0 (0.0%)	0 (0.0%)
Hypotension before administration (systolic BP <90 mmHg)	2 (0.6%)	0 (0.0%)	1 (0.3%)
Known drug allergy	0 (0.0%)	0 (0.0%)	1 (0.3%)

CCT, cardiac computed tomography; HR, heart rate; LVEF, left ventricular ejection fraction; BP, blood pressure.

**Table 2:** **Patient characteristics**

	Low-dose landiolol	High-dose landiolol	Propranolol	P value
	(n = 188)	(n = 213)	(n = 277)	
Age	67.6 ± 10.9	67.7 ± 11.1	67.0 ± 11.9	0.76
Male gender	104 (55.3%)	120 (56.3%)	155 (56.0%)	0.98
Height, cm	159.3 ± 8.5	159.3 ± 9.2	159.7 ± 9.2	0.90
Weight, kg	60.6 ± 11.3	60.5 ± 11.9	61.4 ± 12.9	0.67
<70 kg	149 (79.3%)	168 (78.9%)	215 (77.6%)	0.90
≥70 kg	39 (20.7%)	45 (21.1%)	62 (22.4%)	
Body mass index	23.8 ± 3.3	23.7 ± 3.4	23.9 ± 3.8	0.73
HR before β-blocker, min^-1^	72.9 ± 7.3	72.1 ± 7.7	72.5 ± 7.7	0.61
<75/min	111 (59.0%)	121 (56.8%)	174 (62.8%)	0.39
≥75/min	77 (41.0%)	92 (43.2%)	103 (37.2%)	

HR, heart rate.

Before β-blocker administration, the HR was >75/min in 77 (41.0%) patients from the low-dose landiolol group and 92 (43.2%) patients from the high-dose landiolol group. The mean doses of landiolol in the low-dose and high-dose landiolol groups were 7.7 ± 1.4 mg and 9.2 ± 2.2 mg, respectively. As the landiolol dose was limited up to12.5 mg, 2 patient with low-dose group and 7 patients with high-dose group received 1.75 ± 0.18 mg and 1.36 ± 0.78 mg fewer than prespecified dose, respectively. In the propranolol group, 10 mg of propranolol was administered to 215 patients (77.6%) and a dose of 20 mg was administered to 62 patients (22.4%) (Figure 1).

### Low-dose landiolol versus propranolol

**Figure 3:**
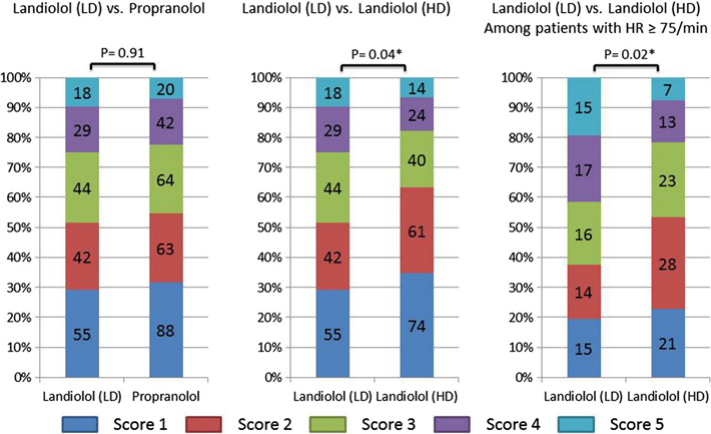
**Image quality scores for all patients and those with an HR ≥75/min before β-blocker administration.** LD, low dose; HD, high dose.

**Figure 4:**
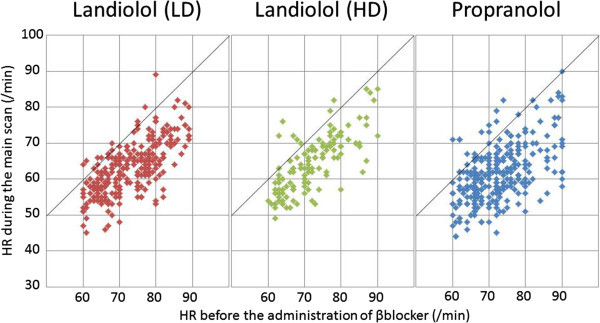
**Average heart rate before β-blocker administration and during the main scan in the low-dose landiolol group, the high-dose landiolol group, and the propranolol group.** HR indicated heart rate; LD, low dose; HD, high dose.

Although the average HR during the main scan was significantly lower in the propranolol group (61.6 ± 8.0/min) compared with that in the patients receiving low-dose landiolol (64.1 ± 7.4/min, P < 0.001), the maximum image quality score was similar between the patients given propranolol (mean 2.43 ± 1.27) and those given low-dose landiolol (mean 2.54 ± 1.31, P = 0.91) (Table 3 and Figure 3). Regarding CT scanning parameters, a higher helical pitch was selected in the propranolol group (P < 0.001). Consequently, multisector reconstruction was employed in 49.1% of the patients from the propranolol group versus 67.7% of those receiving low-dose landiolol. The total radiation dose was smaller in the propranolol group compared with that in the patients receiving low-dose landiolol (1,343 ± 402 DLP versus 1,450 ± 335 DLP, P = 0.003). The decrease of HR was significantly larger in the propranolol group (11.0 ± 7.2/min) compared with that in the patients receiving low-dose landiolol (8.8 ± 5.7/min) (P < 0.001). However, the variance of the decrease in HR was significantly larger in the propranolol group, as shown in Figure 4 (32.5 versus 51.8, P < 0.001). In this study, we assessed HR variability during the scan from the range of HR values, and found no significant difference in the range of HR during the main scan between patients treated with low-dose landiolol group and those given propranolol.

**Table 3:** **Heart rate parameters, CCT scan parameters, and image quality in patients receiving low-dose landiolol or propranolol**

	Low-dose landiolol	Propranolol	P value
	(n = 188)	(n = 277)	
HR before β-blocker, min^-1^	72.9 ± 7.3	72.5 ± 7.7	0.64
Average HR during the main scan, min^-1^	64.1 ± 7.4	61.6 ± 8.0	<0.001
Range of HR during the main scan, min^-1^	5.9 ± 14.3	4.4 ± 10.1	0.19
Decrease of HR, min^-1^	8.8 ± 5.7	11.0 ± 7.2	<0.001
Time between β-blocker administration and main scan, min	4.0 ± 0.6	90.5 ± 16.0	<0.001
Helical pitch			<0.001
Step and shoot	0 (0.0%)	5 (1.8%)	
0.16	138 (73.4%)	141 (50.9%)	
0.18	12 (6.4%)	26 (9.4%)	
0.2	0 (0.0%)	3 (1.1%)	
0.22	4 (2.1%)	22 (7.9%)	
0.24	34 (18.1%)	80 (28.9%)	
No. of sectors used			<0.001
1	61 (32.5%)	141 (50.9%)	
2	119 (63.3%)	125 (45.1%)	
3	8 (4.4%)	11 (4.0%)	
Total exposure, DLP	1450 ± 335	1343 ± 402	0.003
Main scan duration, sec	6.8 ± 1.1	6.4 ± 1.3	<0.001
Premature beats during the main scan	16 (8.5%)	15 (5.4%)	0.19
Failure at breathholding	3 (1.6%)	9 (3.2%)	0.27
Presence of coronary artery plaque	106 (56.3%)	156 (56.3%)	0.99
Presence of calcification	139 (73.9%)	199 (71.8%)	0.62
Maximum image quality score per patient			0.91
1	55 (29.3%)	88 (31.8%)	
2	42 (22.3%)	63 (22.7%)	
3	44 (23.4%)	64 (23.1%)	
4	29 (15.4%)	42 (15.2%)	
5	18 (9.6%)	20 (7.2%)	
Image quality score per vessel			
RCA			0.86
1	67 (35.6%)	106 (38.3%)	
2	40 (21.3%)	66 (23.8%)	
3	38 (20.2%)	50 (18.1%)	
4	31 (16.5%)	40 (14.4%)	
5	12 (6.4%)	15 (5.4%)	
LAD			0.70
1	87 (46.3%)	132 (47.7%)	
2	42 (22.3%)	72 (26.0%)	
3	38 (20.2%)	45 (16.2%)	
4	16 (8.5%)	19 (6.9%)	
5	5 (2.7%)	9 (3.2%)	
LCX			0.32
1	82 (43.6%)	129 (46.6%)	
2	44 (23.4%)	69 (24.9%)	
3	31 (16.5%)	49 (17.7%)	
4	24 (12.8%)	19 (6.9%)	
5	7 (3.7%)	11 (4.0%)	

CCT, cardiac computed tomography; HR, heart rate; DLP, dose-length product; RCA, right coronary artery; LAD, left anterior descending coronary artery; LCX, left circumflex artery.

### Low-dose versus high-dose landiolol

Among patients with a baseline HR ≥75/min (77 [41.0%] patients in the low-dose landiolol group and 92 [43.2%] patients in the high-dose landiolol group), the average HR during the main scan was tended to be lower in the high-dose landiolol group (67.2 ± 6.9/min) compared with that in the low-dose landiolol group (69.0 ± 6.9/min, P = 0.10), and there was a corresponding difference of the maximum image quality score between the high-dose landiolol group (mean 2.53 ± 1.21) and low-dose landiolol group (mean 3.04 ± 1.41, P = 0.02) (Table 4 and Figure 3).

**Table 4:** **Heart rate parameters, CCT scan parameters, and image quality in the low-dose landiolol group and the high-dose landiolol group among patients with baseline HR ≥75/min**

	Low-dose landiolol	High-dose landiolol	P value
	(n = 77)	(n = 92)	
HR before β-blocker, min^-1^	80.3 ± 4.3	79.7 ± 4.1	0.42
Average HR during the main scan, min^-1^	69.0 ± 6.9	67.2 ± 6.9	0.10
Range of HR during the main scan, min^-1^	5.3 ± 12.2	5.9 ± 14.9	0.77
Decrease at HR, min^-1^	11.2 ± 6.0	12.5 ± 6.1	0.18
Time between β-blocker administration and main scan, min	4.1 ± 0.66	3.7 ± 0.80	< 0.001
Helical pitch			0.69
Step and shoot	0 (0.0%)	0 (0.0%)	
0.16	72 (93.5%)	86 (93.5%)	
0.18	3 (3.9%)	5 (5.4%)	
0.2	0 (0.0%)	0 (0.0%)	
0.22	0 (0.0%)	0 (0.0%)	
0.24	2 (2.6%)	1 (1.1%)	
No. of sectors used			0.27
1	10 (13.0%)	6 (6.5%)	
2	59 (76.6%)	79 (85.9%)	
3	8 (10.4%)	7 (7.6%)	
Total exposure, DLP	1582 ± 212	1594 ± 232	0.74
Main scan duration, sec	7.2 ± 0.63	7.5 ± 0.97	0.01
Premature beats during the main scan	6 (7.8%)	6 (6.5%)	0.75
Failure at breathholding	1 (1.3%)	2 (2.2%)	0.67
Presence of coronary artery plaque	32 (41.6%)	51 (55.4%)	0.07
Presence of calcification	53 (68.3%)	73 (79.4%)	0.12
Maximum image quality score per patient			0.02
1	15 (19.5%)	21 (22.8%)	
2	14 (18.2%)	28 (30.4%)	
3	16 (20.8%)	23 (25.0%)	
4	17 (22.1%)	13 (14.1%)	
5	15 (19.5%)	7 (7.6%)	
Image quality score per vessel			
RCA			0.005
1	19 (24.7%)	30 (32.6%)	
2	11 (14.3%)	26 (28.3%)	
3	16 (20.8%)	20 (21.7%)	
4	21 (27.3%)	10 (10.9%)	
5	10 (13.0%)	6 (6.5%)	
LAD			0.054
1	29 (37.7%)	39 (42.4%)	
2	13 (16.9%)	32 (34.8%)	
3	19 (24.7%)	11 (12.0%)	
4	13 (16.9%)	8 (8.7%)	
5	3 (3.9%)	2 (2.2%)	
LCX			0.02
1	29 (37.7%)	44 (47.8%)	
2	12 (15.6%)	20 (21.7%)	
3	10 (13.0%)	17 (18.5%)	
4	19 (24.7%)	8 (8.7%)	
5	7 (9.1%)	3 (3.3%)	

CCT, cardiac computed tomography; HR, heart rate; DLP, dose-length product; RCA, right coronary artery; LAD, left anterior descending coronary artery; LCX, left circumflex artery.

Possible side effects of β-blockers were reported in two patients from the low-dose landiolol group: One patient had sinus bradycardia (40/minute) with frequent ventricular premature beats that recovered after 5 minutes, and the other showed a decrease of blood pressure from 159/95 mmHg to 86/55 mmHg that returned to 114/78 mmHg after 10 minutes. Both patients were asymptomatic.

## Discussion

The objective of the current study was to clarify whether the bolus injection of ultra-short acting β-blocker could improve the image quality or not. The main findings of this study were as follows: (1) Image quality was similar between propranolol and low-dose landiolol, although the decrease in HR was significantly larger in the propranolol group than in the low-dose landiolol groups. (2) In the patients receiving landiolol with a baseline HR ≥75/min, the higher dose was associated with a lower HR and better image quality. (3) Variance in the decrease of HR was significantly smaller and the mean time from administration to performing the main scan was significantly shorter in the landiolol groups.

Despite promising advances in MDCT, heart rate reduction is still essential for CCT. (Leschka et al. 2006; Shim et al. 2005; Muenzel et al. 2011; Korosoglou et al. 2010; Khan et al. 2011). We compared a single bolus injection of landiolol with oral administration of propranolol, and demonstrated a significantly greater decrease of HR in the propranolol group without improvement of image quality. This might be partly explained by the higher frequency of multisector reconstruction with the slight increase of total exposure in the landiolol groups. With a gantry rotation speed of 350 msec, an HR of 62, 68, or 78/min becomes the cut-off value between half-scan reconstruction and multisector reconstruction when the helical pitch is 0.16, 0.18, and 0.20, respectively. (Ohnesorge et al. 2000; Flohr and Ohnesorge 2001). The current study included patients with an HR of around 60/min at the time of the main scan, and there was a significant difference in the performance of multisector reconstruction that might have improved image quality in the patients receiving low-dose landiolol, despite the small absolute difference of 2.5/min for the HR during the main scan.

Leschka et al. reported that not only the absolute reduction of HR but also a decrease of HR variability is important for achieving adequate image quality. (Leschka et al. 2006). The variance in the decrease of HR was smaller in the landiolol groups compared with the propranolol group along with the shorter time between β-blocker administration and performance of the main scan. We needed to avoid specific heart rates synchronized with gantry rotation, as we were unable to perform multisector reconstruction if the cardiac cycle length was an exact multiple of the gantry rotation speed. For example, if the patient had an HR of 86 (cardiac cycle length = 700 msec) during the main scan, we could not perform multisector reconstruction when the gantry rotation speed was 350 Msec. A protocol that involves continuous infusion with stepwise dose increments might be more beneficial for controlling the HR prior to CCT. However, the current study demonstrated that an additional bolus dose could also reduce the HR and improve image quality without any complications. Further investigation to assess higher-dose bolus injection alone and bolus injection following oral administration of a β-blocker would be needed to clarify the efficacy of bolus landiolol compared with a stepwise continuous infusion protocol.

In this study, two patients developed suspected side effects after bolus injection of landiolol, including transient sinus bradycardia and severe hypotension. Owing to the short half-life of this agent, (Atarashi et al. 2000). The adverse effects were not prolonged and resolved completely. De Graaf et al. reported that contraindications to oral β-blocker therapy exist in a substantial proportion of patients undergoing CCT, (De Graaf et al. 2010). although administration of β-blocker was essential for better image quality. Because the absolute number of patients with adverse events was small, we could not assess the difference in the incidence of events between patients receiving bolus injection of landiolol and those given oral propranolol. However, landiolol is a highly β1-selective and ultra-short acting β-blocker that is expected to be associated with less persistent adverse effects.

**Figure 5:**
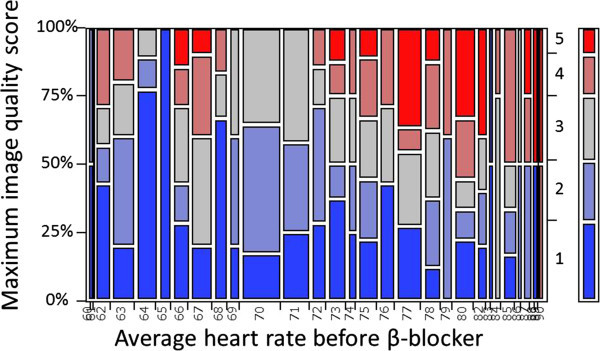
**Mosaic plot for maximum image quality socre and average heart rate before β-blocker in patients with low-dose landiolol group.**

The present study has several limitations. First, the dose of landiolol was specified and additional injections were prohibited by the study protocol. Adjusting the dose of landiolol for each patient could have achieved the optimal HR and further improved image quality. Furthermore, a prespecified dose of 3.75 mg and HR of 75/min would not be most effective cut off points. However, in low-dose landiolol group, patients with the baseline HR ≥ 75/min were prone to have poor image quality (maximum image score ≥ 4) as compared with those with baseline HR < 75/min as shown in Figure 5. Second, this was not a randomized trial, hence the results may be biased. Because the helical pitch was set at the discretion of the radiologist, there were substantial differences of the CT scanning parameters and post-processing. Although these parameters were mainly determined automatically based on the HR just before the main scan, the radiologists expected that the HR would change after scanning parameters were selected in the landiolol groups. Third, the number of patients was relatively small (especially for evaluating safety), even though more than 400 patients were treated with landiolol.Fourth, the low penetration rate of step and shoot mode use could increase the radiation dose. Furthermore, radiation dose did not decrease despite of injection of high dose landiolol. Using step and shoot technique could be more beneficial not only for better image quality but also for reducing radiation dose. Finally, we did not evaluate the other medications prior to the CCT except for the β-blocker based on the study protocol. Administrating the β-blocker could not be effective in patients with prior medication of cardiovascular drugs including β-blocker for coexisting cardiac disease.

## Conclusions

Although the decrease of HR was significantly larger in the propranolol group than in the landiolol groups, the image quality was similar with the two β-blockers. Among the patients receiving landiolol, a higher dose was associated with a lower HR and better image quality. Further investigation to assess higher-dose bolus injection of landiolol or bolus injection following oral administration of a β-blocker would be needed.

## Abbreviations

CCT: Cardiac computed tomography
HR: Heart rate
MDCT: Multidetector computed tomography.
